# Scope for non-crop plants to promote conservation biological control of crop pests and serve as sources of botanical insecticides

**DOI:** 10.1038/s41598-020-63709-x

**Published:** 2020-04-24

**Authors:** B. W. Amoabeng, P. C. Stevenson, B. M. Mochiah, K. P. Asare, G. M. Gurr

**Affiliations:** 1 School of Agriculture and Wine Sciences, Charles Sturt University, Orange Campus, Orange, New South Wales Australia; 2 Council for Scientific and Industrial Research (CSIR), Crops Research Institute, Kumasi, Ghana; 3 Natural Resources Institute, University of Greenwich, Kent, ME4 4TB United Kingdom; 4 Royal Botanic Gardens, Kew, Surrey TW9 3AB United Kingdom; 5 Department of Mathematics and Statistics, Kumasi Technical University, Kumasi, Ghana; 6 Graham Centre for Agricultural Innovation (New South Wales Department of Primary Industries and Charles Sturt University), Orange, New South Wales Australia; 7 Institute of Applied Ecology, Fujian Agriculture & Forestry University, Fuzhou, Fujian China

**Keywords:** Agroecology, Plant ecology

## Abstract

Besides providing food and shelter to natural enemies of crop pests, plants used in conservation biological control interventions potentially provide additional ecosystem services including providing botanical insecticides. Here we concurrently tested the strength of these two services from six non-crop plants in managing cabbage pests in Ghana over three successive field seasons. Crop margin plantings of *Ageratum conyzoides, Tridax procumbens*, *Crotalaria juncea, Cymbopogon citratus, Lantana camara* and *Talinum triangulare* were compared with a bare earth control in a three-way split plot design such that the crop in each plot was sprayed with either a 10% (w/v) aqueous extract from the border plant species, a negative control (water) and a positive control (emamectin benzoate ‘Attack’ insecticide). Pests were significantly less numerous in all unsprayed treatments with non-crop plant margins and in corresponding sprayed treatments (with botanical or synthetic insecticide positive control) while treatments with bare earth margin or sprayed with water (negative controls) had the highest pest densities. Numbers of predators were significantly depressed by synthetic insecticide but higher in other treatments whether unsprayed or sprayed with botanical insecticide. We conclude that some plant species have utility in both conservation biological control and as source of botanical insecticides that are relatively benign to natural enemies. In this crop system, however, the additional cost associated with using botanical insecticides was not justified by greater levels of pest suppression than achieved from border plants alone.

## Introduction

Insect pest attack causes huge crop losses^1^ affecting the potential availability of food for over one billion people^2^, and threatening global food security^3^. Effective pest management is critical to meeting global food demands^4–6^ and synthetic insecticides have been the principal tool in the past six decades^7,8^. Even with the introduction of newer and relatively safer insecticides^8^ and increased application per unit area and time^9^, crop loss resulting from insect damage has doubled in the past four decades^1,10,11^. Indiscriminate and excessive application has led to the elimination of important organisms in the agroecosystem that support crop production^12,13^. This is especially prevalent in developing countries including Ghana where farmers and the environment are exposed to high levels of synthetic insecticides, including active ingredients that have been banned in developed nations due to high toxicity and persistence^14^. Inadequate provision of important ecosystem services including natural enemy-mediated pest control reinforces reliance on hazardous inputs such as insecticides and threatens agricultural sustainability^15,16^.

Conservation biological control based on habitat manipulation has been proposed as a sustainable alternative to synthetic insecticides^17^. Habitat manipulation involves intentionally establishing plant species at the farm scale or landscape scale to provide conducive habitats, floral resources and alternative prey at the right time and space to support natural enemy assemblages to promote biological pest suppression^18,19^. This approach to conservation biological control can result in other benefits besides insect-mediated pest suppression, such as pollination^15^ and provisioning botanical insecticides, herbal medicines, spices and indigenous food^20,21^. Insecticides obtained from plants (‘botanicals’) have been documented to be cost-effective and sustainable in managing insect pests including those that have developed resistance to synthetic insecticides^22–25^. Botanical insecticides cause minimal harm to predators and parasitoids because they usually exhibit actions such as repellence, anti-feeding, oviposition deterrence rather than toxicity^26^. Whilst botanicals can exhibit toxicity, they tend to pose minimal environmental impact when used in the crude form at low concentrations^26^ as when used as ‘home-made’ products by smallholder farmers. Furthermore, botanicals break down easily upon exposure to air and sunlight, so are more compatible with ecologically-based pest management systems such as conservation biological control^27–29^.

Integrated pest management (IPM) combines various pest management tactics as much as practicable to maintain pest population below the economic injury level in a sustainable manner^4,30^. The integration of two or more pest management tactics may deliver better long term pest suppression than one tactic in isolation^30^. However, the combination of the various tactics must not result in major trade-offs or have lethal and/or sub-lethal effects on non-target organisms especially natural enemies that form the core of conservation biological control^16,31^.

Although both conservation biological control^18,19^ and botanicals^23,24,32,33^ have been successfully used in managing field pests in separate applications, combining the two tactics requires careful assessment of their respective and combined effects on pest and natural enemies^34^ as well as the overall impacts on crop yield and quality. In addition, there is an expected increased cost of plant protection with two pest management tactics and thus, an economic analysis must be done to ensure the viability and profitability for such interventions.

A number of studies have explored the effect of synthetic insecticides on biological control agents and report varying effects on natural enemies e.g.^34–36^. Yet, few studies have reported on impacts of synthetic insecticides on both pests and natural enemies as well as on crop yield e.g.^37,38^. To date, the value of conservation biological control especially where it is integrated with pesticides is unclear^38^ and no study has either combined habitat manipulation for conservation biological control and the use of botanicals, or analyzed the economic implications of combining these tools.

We address this gap by studying over three consecutive seasons the effects of six non-crop plant species that are native or naturalized in Ghana and readily available to smallholder farmers. *Ageratum conyzoides*, *Tridax procumbens* (Asteraceae), *Crotalaria juncea* (Fabaceae), *Cymbopogon citratus* (Poaceae), *Lantana camara* (Verbenaceae) and *Talinum triangulare* (Talinaceae) were tested in field experiments. Each was used as a crop margin habitat manipulation plant to promote conservation biological control by providing floral resources as well as shelter for natural enemies and source of botanicals in a factorial design to quantify the combined and individual effects of botanicals and conservation biological control on three major pests of cabbage, diamondback moth, *Plutella xylostella* L. (Lepidoptera: Plutellidae), cabbage aphid, *Brevicoryne brassicae* L. (Hemiptera: Aphididae) and cabbage web worm, *Hellula undalis* Fab. (Lepidoptera: Crambidae) as well as two predators, spiders (Araneae) and ladybird beetles (Coccinellidae).

## Results

### Effects of habitat manipulation and spraying on *P. xylostella*

In all the three seasons, habitat manipulation had significant (P < 0.05) effects on mean numbers of *P. xylostella* per plant. All treatments with habitat manipulation plants were significantly better than the no plant in all weeks (Tables 1, 2 and 3). Both botanicals and Attack were not different from each other in season one but were both significantly lower in *P. xylostella* numbers than the tap water control (Table 1). In week one of season two, botanicals significantly reduced *P. xylostella* numbers better than both Attack and tap water which were not different from each other (Table 2). However, in week two, botanicals and Attack performed equally but both were significantly better than the tap water control. In week three, Attack reduced *P. xylostella* numbers significantly better than both botanicals and tap water which were not different from each other. In week four, Attack as significantly better than both botanicals and tap water whilst botanical was also significantly better than the tap water (Table 2). In week two and four of season three, botanicals and Attack were not significantly different from each other but were both better than the tap water (Table 3). But in week one, botanicals gave significantly lower numbers of *P. xylostella* compared with both Attack and tap water which were not significantly different from each other. However, in week three, Attack was significantly lower than both botanicals while botanicals were also better than the tap water with mean *P. xylostella* numbers of 0.18, 0.46 and 0.79 per plant for Attack^®^, botanicals and tap water respectively (Table 3).

**Table 1:** Main effects of habitat manipulation and spraying on mean numbers of *P. xylostella* in season one (June to August, 2017) in Kumasi, Ghana.

Treatment	Week 1	Week 2	Week 3	Week 4	N
**Habitat manipulation**					
*Ageratum conyzoides*	0.08b	0.17b	0.00b	0.00c	12
*Crotalaria juncea*	0.17b	0.25b	0.25b	0.42b	12
*Cymbopogon citratus*	0.14b	0.25b	0.00b	0.00c	12
*Lantana camara*	0.02b	0.17b	0.00b	0.00c	12
*Talinum triangulare*	0.00b	0.08b	0.00b	0.00c	12
*Tridax procumbens*	0.08b	0.33b	0.00b	0.00c	12
No plant	0.63a	1.00a	0.87a	1.00a	12
P	0.000	0.009	0.001	0.001	12
F	5.28	4.42	20.61	12.42	8
Df	6,60	6,60	6,60	60,60	
**Spraying**					
Botanical	0.05b	0.17b	0.00b	0.08b	24
Attack®	0.04b	0.18b	0.04b	0.07b	28
Water	0.29a	0.50a	0.32b	0.32a	28
P	0.003	0.016	0.000	0.019	
F	6.12	4.42	14.46	4.32	
Df	2,60	2,60	2,20	2,60

**Table 2:** Main effects of habitat manipulation and spraying on mean numbers of *P. xylostella* in season two (September to November 2017) in Kumasi, Ghana.

Treatment	Week 1	Week 2	Week 3	Week 4	N
**Habitat manipulation**					
*Ageratum conyzoides*	0.25b	0.00c	0.42b	0.75bc	12
*Crotalaria juncea*	0.17b	0.58b	0.67b	0.67bc	12
*Cymbopogon citratus*	0.17b	0.00c	0.50b	0.92b	12
*Lantana camara*	0.25b	0.33bc	0.25b	0.42bc	12
*Talinum triangulare*	0.00b	0.00c	0.17b	0.17c	12
*Tridax procumbens*	0.00b	0.00c	0.58b	0.50bc	12
No plant	1.25a	1.13a	1.75a	2.00a	8
P	0.001	0.001	0.001	0.001	
F	18.26	23.54	14.22	15.42	
Df	6,60	6,60	6,60	6,60	
**Spraying**					
Botanical	0.00b	0.20b	0.63a	0.70b	24
Attack®	0.35a	0.10b	0.18b	0.36c	28
Water	0.36a	0.43a	0.89a	1.07a	28
P < 0.05	0.013	0.001	0.001	0.001	
F	4.63	10.38	22.68	18.44	
Df	2,60

**Table 3:** Main effects of habitat manipulation and spraying on mean numbers of *P. xylostella* in season two (December 2017 to March 2018) in Kumasi, Ghana.

Treatment	Week 1	Week 2	Week 3	Wek 4	N
**Habitat manipulation**					
*Ageratum conyzoides*	0.33b	0.00c	0.42b	0.58b	12
*Crotalaria juncea*	0.17b	0.50b	0.50b	0.58b	12
*Cymbopogon citratus*	0.17b	0.00c	0.42b	0.58b	12
*Lantana camara*	0.25b	0.33bc	0.17b	0.33b	12
*Talinum triangulare*	0.00b	0.00c	0.08b	0.17b	12
*Tridax procumbens*	0.00b	0.00c	0.42b	0.33b	12
No plant	1.13a	1.00a	1.75a	1.75a	8
P	0.001	0.001	0.001	0.001	
F	14.61	15.36	15.88	11.82	
Df	6,60	6,60	6,60	6,60	
**Spraying**					
Botanical	0.00b	0.20ab	0.46b	0.50b	24
Attack®	0.36a	0.07b	0.18c	0.25b	28
Water	0.36a	0.39a	0.79a	0.93a	28
P	0.001	0.001	0.001	0.001	
F	5.60	8.84	16.35	16.21	
Df	2,60	2,60	2,60	2,60	

### Effects of habitat manipulation and spraying on *B. brassicae*

Habitat manipulation had a significant effect on *B. brassicae* infestation severity in week one, three and four in season one. Habitat manipulation with *T. procumbens* was significantly lower (P = 0.011, F = 3.05.68, df = 6, 60) in *B. brassica*e score than habitat manipulation with *A. conyzoides* and the no plant control but not different from the remaining treatments. In week three, all habitat manipulation treatments with plants were significantly lower (P = 0.001, F = 13.12, df = 6,60) in *B. brassicae* score than the no plant control treatment. Habitat manipulation with *T. procumbens* and *C. juncea* were lower in *B. brassicae* than all other treatments. Spraying had an effect only in week three where Attack was significantly (P = 0.001, F = 13.96, df = 2,60) lower than botanical while botanical was significantly lower than tap water in *B. brassicae* score. Habitat manipulation had significant effect of *B. brassicae* score in all weeks in season two but no effect of spraying was observed. In week one, the no habitat manipulation had significantly higher (P = 0.032, F = 2.48, df = 6,60) *B. brassicae* score than the remaining treatments except habitat manipulation with *L. camara* and *C. citratus*. All habitat manipulation treatments with non-crop strips were significantly (P = 0.001, F = 5.44, df = 6,60) lower in *B. brassicae* score than the no plant manipulation except plots with *C. juncea* and *L. camara*. In weeks three and four, the no plant control was higher in *B. brassicae* numbers than the habitat manipulation treatments with plant strips. No effects of habitat manipulation and spraying were observed throughout season three.

Effect of both habitat manipulation and spraying on *H. undalis* were found only in the first week of season two. Habitat manipulation with *C. juncea, C. citratus* and *T. triangulare* were significantly (P = 0.001, F = 4.15, df = 6,60) lower than the remaining treatments.

From Table 4, there were significant interactions between week and both habitat manipulation and spraying and necessitated Tukey’s post-hoc (HSD) to differentiate which weeks combined with the factor levels differ from each other.

**Table 4:** Overall analysis of variance of interaction between week and both habitat manipulation and spraying.

Effect		Value	F	Hypothesis df	Error df	Sig.	Partial Eta Squared
Week	Wilks’ Lambda	0.003	5639.362^b^	3.000	58.000	0.000	0.997
Week*Habitat manipulation	Wilks’ Lambda	0.021	26.394	18.000	164.534	0.000	0.722
Week*Spraying	Wilks’ Lambda	0.456	9.284^b^	6.000	116.000	0.000	0.324
	Wilks’ Lambda	0.122	5.410	33.000	171.583	0.000	0.504

**Figure 1:**
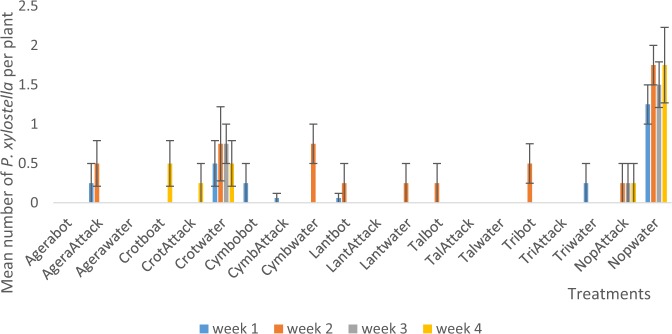
Mean (SE±) *P. xylostella* numbers in interaction between habitat manipulation and spraying in season one (June to August, 2017) in Kumasi Ghana.

**Figure 2:**
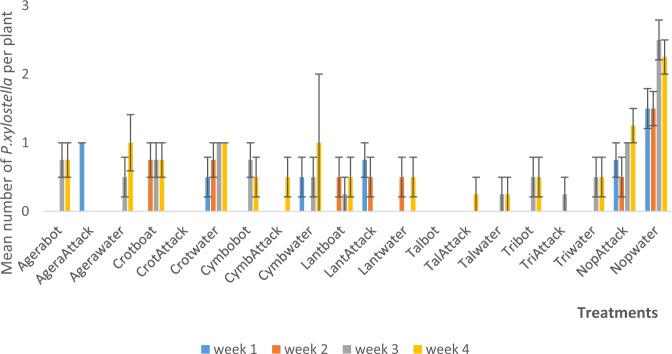
Mean (SE±) *P. xylostella* numbers in interaction between habitat manipulation and spraying in season two (September to November, 2017) in Kumasi Ghana.

**Figure 3:**
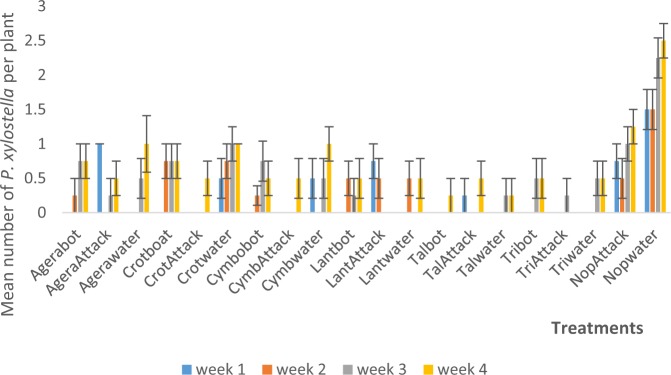
Mean (SE±) *P. xylostella* numbers in interaction between habitat manipulation and spraying in season three (December 2017 to March, 2018) in Kumasi Ghana.

Interaction between habitat manipulation and spraying resulted in a significant (P < 0.05) effect on numbers of *P. xylostella*. The no plant, water-sprayed (‘Nopwater’) treatment was significantly (P < 0.05) higher in numbers of *P. xylostella* in all seasons (Fig. 1), two (Fig. 2) and three (Fig. 3) except in week four of season two where it was not significantly different from the Crotalaria-bordered, water-sprayed (‘Crotwater’) treatment.

**Figure 4:**
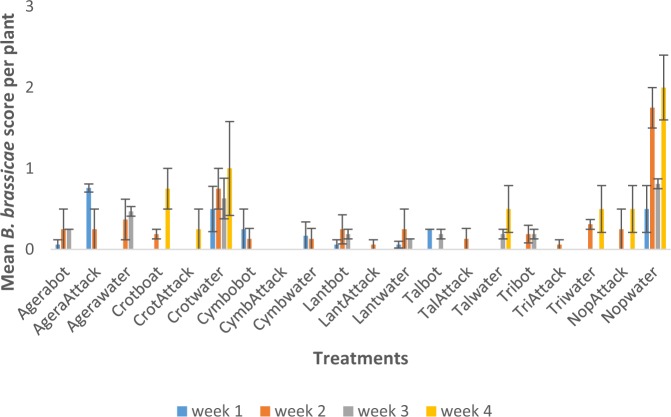
Mean (SE±) *B. brassicae* numbers in interaction between habitat manipulation and spraying in season one (June to August, 2017) in Kumasi Ghana.

**Figure 5:**
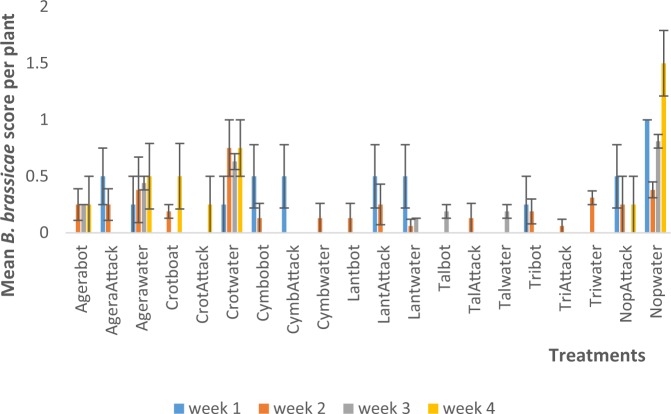
Mean (SE±) *B. brassicae* numbers in interaction between habitat manipulation and spraying in season one (September to November, 2017) in Kumasi Ghana.

The Ageratum-bordered, synthetic insecticide (Attack)-sprayed (‘AgeraAttack’) treatment combination was significantly (P < 0.05) higher in *B. brassicae* score in week one than the remaining treatments except Crotwater and Nopwater. In week two, the Nopwater treatment was significantly higher than the remaining treatments (Fig. 4). In season two week one, Nopwater was significantly higher than the remaining treatments in *B. brassicae* infestation score except in week two when it was not higher than Crotwater (Fig. 5).

### Effect of treatments on predators

**Figure 6:**
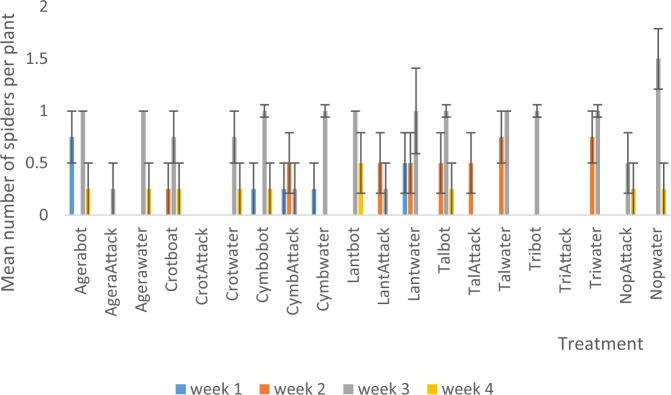
Mean (SE±) of spiders numbers in interaction between habitat manipulation and spraying in season two (September to November, 2017) in Kumasi Ghana.

**Figure 7:**
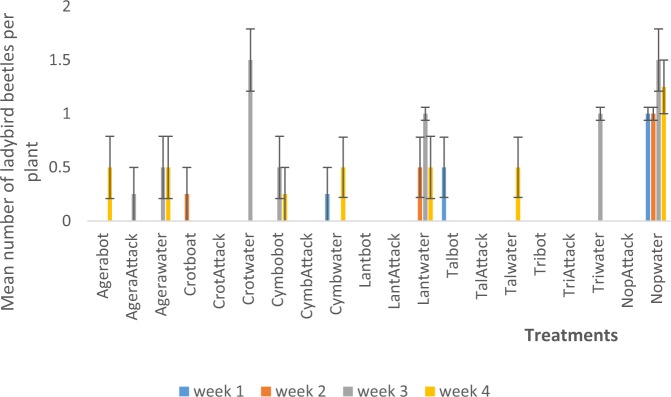
Mean (SE±) of ladybird beetles numbers in interaction between habitat manipulation and spraying in season one (June to August, 2017) in Kumasi Ghana.

Interaction between habitat manipulation and spraying resulted in a significant effect only in week three of season two in which the Nopwater was significantly (P < 0.05) higher in spider numbers than the remaining treatments (Fig. 6). In season one Nopwater was significantly (P < 0.05) higher in ladybird beetle numbers than the remaining treatment combinations (Fig. 7).

### Effect of treatments on cabbage head yield and quality

There were significant interactions between habitat manipulation and spraying. In season one, the highest yield per plant was observed in LanAttack treatment but was not significantly (P > 0.05) better than any of the habitat manipulation and botanical insecticide combinations except Agerabot (Table 5). In season two, TalAttack had the highest yield per plant and was better than eight treatments including Nopwater. In season three, Talwater had the highest yield per plant and was significantly (P < 0.05) better than only one (Crotbot) habitat manipulation and botanical insecticide combinations, four habitat manipulations plus Attack combinations, four habitat manipulations only treatments and the no habitat manipulation treatment (Nopwater) (Table 5). NopAttack had the highest yield per hectare in season one and was significantly (P < 0.05) better the remaining treatments except LanAttack, Lanbot, Cymbot and Lanwater (Table 6). The Nopwater had the highest damaged yield per hectare in seasons one and two. It was significantly (P < 0.05) higher than the remaining treatments except in season two when it was not significantly different from Crotwater (Table 6). TalAttack was the highest in undamaged yield per hectare in season two and was significantly (P < 0.05) better than ten treatments including the Nopwater which had the lowest undamaged yield per hectare (Table 6). In season three, Lanwater was the highest in yield per hectare and was significantly (P < 0.05) better than nine treatments including the Nopwater (Table 6).

**Table 5:** Mean (±SE) effect of habitat manipulation and spraying on cabbage yield per plant in three season in Kumasi, Ghana.

Treatment	Season one (Kg/plant)	Season two (Kg/plant)	Season three (Kg/plant)
Agerabot	0.64 ± 0.02bc	0.83 ± 0.01ab	0.85 ± 0.04abcd
AgeraAttack	0.62 ± 0.01bcd	0.60 ± 0.04def	0.73 ± 0.02bcde
Agerawater	0.52 ± 0.01cd	0.74 ± 0.04abcd	0.69 ± 0.02de
Crotbot	0.67 ± 0.05abc	0.63 ± 0.02cdef	0.72 ± 0.01cde
CrotAttack	0.54 ± 0.04cd	0.65 ± 0.01bcdef	0.68 ± 0.07de
Crotwater	0.52 ± 0.01cd	0.80 ± 0.04abc	0.70 ± 0.04cde
Cymbot	0.76 ± 0.03ab	0.59 ± 0.04ef	0.79 ± 0.01abcde
CymAttack	0.73 ± 0.01ab	0.66 ± 0.02bcdef	0.75 ± 0.03abcde
Cymwater	0.57 ± 0.02cd	0.61 ± 0.02def	0.75 ± 0.03abcde
Lanbot	0.73 ± 0.03ab	0.73 ± 0.04abcde	0.80 ± 0.02abcde
LanAttack	0.78 ± 0.03a	0.64 ± 0.05cdef	0.84 ± 0.02abcd
Lanwater	0.74 ± 0.03ab	0.69 ± 0.02abcdef	0.77 ± 0.06abcde
Talbot	0.66 ± 0.02abc	0.78 ± 0.03abcd	0.90 ± 0.01ab
TalAttack	0.66 ± 0.03abc	0.85 ± 0.05a	0.87 ± 0.02abc
Talwater	0.54 ± 0.02cd	0.73 ± 0.06abcde	0.91 ± 0.01a
Tribot	0.66 ± 0.06abc	0.61 ± 0.06def	0.85 ± 0.01abcd
TriAttack	0.73 ± 0.03ab	0.80 ± 0.01abc	0.66 ± 0.02e
Triwater	0.56 ± 0.02cd	0.65 ± 0.01bcdef	0.78 ± 0.01abcde
NopAttack	0.77 ± 0.01ab	0.67 ± 0.02abcdef	0.73 ± 0.06bcde
Nopwater	0.48 ± 0.04d	0.52 ± 0.02f	0.66 ± 0.03e
P	0.001	0.001	0.001
F	10.08	6.60	5.73
df	11, 60	11, 60	11, 60

Means within a column with different letters differ significantly (P < 0.05).

**Table 6:** Effects of habitat manipulation and spraying on yield and quality of cabbage in three seasons in Kumasi, Ghana.

Treatment	Season one		Season two		Season three
	Undamaged yield (t/ha)	Damaged yield (t/ha)	Undamaged yield (t/ha)	Damaged yield (t/ha)	Undamaged yield (t/ha)
Agerabot	19.96 ± 1.13bcde	2.44 ± 0.89bcd	27.05 ± 0.51a	2.00 ± 0.16ef	33.25 ± 1.34abc
AgeraAttack	19.96 ± 0.45bcde	1.74 ± 0.66bcd	19.15 ± 1.49cdef	1.85 ± 0.41ef	28.57 ± 0.83bcde
Agerawater	14.79 ± 0.81e	3.41 ± 0.97bc	22.50 ± 1.89bcdef	3.40 ± 0.54bc	26.81 ± 1.22e
Crotbot	21.74 ± 1.64bcde	1.71 ± 0.43bcd	19.72 ± 0.98cdef	2.33 ± 0.081ef	28.28 ± 0.51bcde
CrotAttack	17.97 ± 1.32de	0.93 ± 0.09cd	19.47 ± 0.52cdef	3.28 ± 0.11bcd	26.62 ± 2.51e
Crotwater	17.42 ± 0.29de	0.78 ± 0.16cd	23.22 ± 1.53bcdef	4.78 ± 0.13a	27.20 ± 1.40de
Cymbot	22.59 ± 0.63bcd	4.01 ± 0.28b	18.70 ± 1.44f	1.95 ± 0.09ef	30.71 ± 0.52abcde
CymAttack	22.27 ± 0.77bcde	3.28 ± 0.32bc	19.62 ± 1.03cdef	3.48 ± 0.14bcd	28.25 ± 0.92bcde
Cymwater	17.28 ± 0.37de	2.67 ± 0.71bcd	18.52 ± 0.78f	2.83 ± 0.11bcde	27.20 ± 0.60de
Lanbot	24.41 ± 0.77abc	1.14 ± 0.26bcd	23.92 ± 1.48bcde	1.63 ± 0.16f	32.86 ± 0.71abcde
LanAttack	24.89 ± 1.23ab	2.41 ± 0.42bcd	20.67 ± 1.80cdef	1.73 ± 0.08f	32.86 ± 0.66abcd
Lanwater	22.63 ± 0.95bcd	3.27 ± 0.67bc	21.92 ± 0.38bcdef	2.23 ± 0.09ef	29.84 ± 2.07abcde
Talbot	21.99 ± 1.10bcde	1.11 ± 0.24cd	24.45 ± 0.92abc	2.85 ± 0.12cde	35.10 ± 0.38a
TalAttack	22.91 ± 0.88abcd	0.19 ± 0.19d	27.30 ± 2.00a	2.75 ± 0.41def	33.83 ± 0.39ab
Talwater	18.22 ± 1.24de	0.68 ± 0.27cd	23.02 ± 2.16bcdef	2.53 ± 0.14def	35.30 ± 0.44a
Tribot	20.58 ± 2.30bcde	2.52 ± 0.63bcd	18.97 ± 1.95def	2.38 ± 0.29ef	32.99 ± 0.59abcd
TriAttack	23.07 ± 1.19abcd	2.48 ± 0.74bcd	25.90 ± 0.47ab	2.10 ± 0.23ef	25.64 ± 0.44e
Triwater	16.37 ± 0.07e	3.23 ± 0.69bc	20.60 ± 0.48ef	2.15 ± 0.09ef	30.52 ± 0.41abcde
NopAttack	26.30 ± 0.94a	1.00 ± 0.65bcd	19.71 ± 0.74bcdef	4.08 ± 0.13ab	29.45 ± 0.63abcde
Nopwater	9.49 ± 1.95f	7.55 ± 0.51a	13.41 ± 0.44g	5.05 ± 0.44a	26.50 ± 0.64e
P	0.001	0.001	0.001	0.001	0.001
F	7.34	12.73	6.53	14.41	6.91
df	11, 60	11, 60	11, 60	11, 60	11, 60

Means within a column with different letters differ significantly (P < 0.05). Note: No damaged yield in season three. Yield per hectare = head weight per plant × plant population per hectare.

### Economic analyses

#### Cost of plant protection and income

Costs of plant protection ranged between $70.40 and $257.40 per hectare (Table 7). The habitat manipulation-only treatments were lowest whereas the habitat manipulation plus Attack treatments were the highest. In season one, the NopAttack had the highest income from undamaged yield and highest net income per hectare while the Nopwater was the lowest in income from undamaged yield per hectare. Among the habitat manipulation treatments, LanAttack was the highest in both incomes from undamaged yield and net income per hectare. In each habitat manipulation combination, the tap water spraying options were the lowest in income from undamaged income (Table 7). In season two, TalAttack was the highest in both incomes from undamaged yield and net income per hectare whilst the lowest was the Nopwater. In season three, Talwater had the highest income from undamaged heads as well as net income per hectare whereas the Nopwater was the lowest in income from undamaged heads but CrotAttack was the lowest in net income per hectare (Table 7).

**Table 7:** Evaluation of cost and benefit of habitat manipulation for conservation biological control and spraying on cabbage pests in three seasons in Kumasi, Ghana.

Treatment	Income from undamaged yield (US$)			Income from damaged yield (US$)		Cost of protection	Net income (US$)			Cost benefit ratio		
	Season 1	Season 2	Season 3	Season 1	Season 2	US$	Season 1	Season 2	Season 3	Season 1	Season 2	Season 3
Agerabot	8,782.40	8,926.50	10,972.50	536.80	440.00	194.60	9,124.60	9,171.90	10,777.60	1: 16.90	1: 18.68	1: 10.45
AgeraAttack	8,782.40	6,319.50	9,428.10	382.80	407.00	231.00	8,934.20	6,495.50	9,197.10	1: 13.41	1: 4.15	1: 1.96
Agerawater	6,507.60	7,425.00	8,847.30	750.20	748.00	70.40	7,187.40	8,102.60	8,776.90	1: 19.19	1: 36.30	1: 0.45
Crotbot	9,565.60	6,507.60	9,332.40	376.20	512.60	212.20	9,729.60	6,808.00	9,120.20	1: 18.32	1: 5.99	1: 1.77
CrotAttack	7,906.80	6,425.10	8,784.60	204.60	721.60	248.60	7,862.80	6,898.10	8,536.00	1: 8.15	1: 5.48	1: −0.84
Crotwater	7,664.80	7,662.60	8,976.00	171.60	1,051.60	88.00	7,748.40	8,626.20	8,888.00	1: 21.73	1: 35.11	1: 1.63
Cymbot	9,939.60	6,171.00	10,134.30	882.20	429.00	212.20	10,609.60	6,387.80	9,922.10	1: 22.49	1: 4.01	1: 5.55
CymAttack	9,798.80	6,474.60	9,322.50	721.60	765.60	248.60	10,235.80	6,991.60	9,073.90	1: 17.70	1: 5.85	1: 1.32
Cymwater	7,603.20	6,111.60	8,976.00	587.40	622.60	88.00	8,102.60	6,646.20	8,888.00	1: 25.75	1: 12.61	1: 1.63
Lanbot	10,740.40	7,893.60	10,843.80	250.80	358.60	221.00	10,769.80	8,031.20	10,622.80	1: 22.32	1: 11.29	1: 8.50
LanAttack	10,951.60	6,821.11	10,843.80	530.20	380.60	257.40	11,224.40	6,944.31	10,586.40	1: 20.93	1: 5.47	1: 7.15
Lanwater	9,957.20	7,233.60	9,847.20	719.40	490.60	96.80	10,579.80	7,627.40	9,750.40	1: 49.00	1: 21.60	1: 10.39
Talbot	9,675.60	8,068.50	11,583.00	244.20	672.00	203.40	9,716.40	8,537.10	11,379.60	1: 19.07	1: 14.75	1: 12.95
TalAttack	10,080.40	9,009.00	11,163.90	41.80	605.00	239.80	9,882.40	9,374.20	10,924.10	1: 16.87	1: 16.01	1: 9.09
Talwater	8,016.80	7596.60	11,649.00	149.60	556.60	79.20	8,087.20	8,074.00	11,569.80	1: 28.42	1: 32.04	1: 35.67
Tribot	9,055.20	6,270.10	10,886.70	554.40	523.60	194.60	9,415.00	6,599.10	10,692.10	1: 18.39	1: 5.46	1: 10.01
TriAttack	10,150.80	8,547.00	8,461.20	545.60	462.00	231.00	10,465.40	8,778.00	8,230.20	1: 20.04	1: 14.03	1: −2.23
Triwater	7,202.80	6,798.00	10,071.60	710.60	473.00	70.40	7,843.00	7,200.60	10,001.20	1: 28.50	1: 23.64	1: 17.84
NopAttack	11,572.00	6,504.30	9,718.50	220.00	897.60	172.64	11,619.40	7,229.26	9,545.86	1: 33.50	1: 9.81	1: 4.64
Nopwater	4,175.60	4,425.30	8,745.00	1,661.00	1,111.00	0.00	5,836.60	5,536.30	8,745.00	—	—	—

Note: All analyses are seasonal based. Cost of plant protection was the same across all season. Income from undamaged yield = total weight of undamaged yield × price (kg) undamaged yield. Income from damaged yield = total weight of damaged yield × price (kg) damaged yield. Total income = income from undamaged yield + income from damaged yield. Net income = Total income – cost of protection (for each treatment). Benefit over control treatment = Net income for each treatment – income from control. Cost: benefit ratio = Benefit over control for each treatment $\div$ cost of protection for each treatment. Economic analysis followed the procedure in Amoabeng *et al* (2014).

#### Cost: benefit ratios

In season one, cost: benefit ratios ranged between 1: 8.15 and 1: 49.00 for CrotAttack and Lanwater respectively. In season two, ratios between 1: 4.01 and 1: 36.3 for Crotbot and Agerawater respectively were obtained while in season three, CrotAttack and TriAttack recorded negative ratios of 1: −0.84 and 1: −2.23 respectively. The highest cost: benefit ratio was obtained in Talwater 1: 35.67 (Table 7).

## Discussion

Both habitat manipulation with the non-crop plants and spraying contributed to pest suppression in this study, with the effects cascading on improved cabbage head yield and quality, overall net income, and cost: benefit ratios in all seasons. The no plant manipulation control sprayed with Attack (‘NopAttack) suppressed pests and resulted in the highest yield in the first season but was subsequently outperformed by some of the habitat manipulation treatments. In contrast, Nopwater showed high numbers of pests resulting in reduced yield and quality of cabbage in all the three seasons. The various non-crop plants exhibited different potential in supporting conservation biological control and as botanical insecticides. Higher cost: benefit ratios were exhibited by the treatments especially habitat manipulation without spraying options. Habitat manipulation with non-crop strips plus spraying with Attack had a strong additive effect in season one but this was reduced in season two and subsequently in trade-offs in season three.

This study demonstrated the potential of exploiting the dual ecosystem services of botanical insecticides and conservation biological control for managing brassicas pests. Habitat manipulation for conservation biological control, however, was more potent in suppressing pests compared with the use of botanicals of the same plant species. Habitat manipulation with most of the plant species sprayed with tap water had lower numbers of pests comparable with habitat manipulation sprayed with botanicals or Attack. For instance, habitat manipulation with *T. triangulare* and spraying with both tap water and botanicals gave low numbers of pests even though aqueous extracts of *T. triangulare* was found to be inactive against *P. xylostella* and *B. brassicae* in an earlier lab bioassay (Amoabeng *et al*., unpublished report). This suggests that natural enemies might be exclusively responsible for pest suppression in those treatments and also indicating the potential of *T. triangulare* in habitat manipulation for conservation biological control. In contrast, habitat manipulation with *C. juncea* and sprayed with botanical from the same plant as well as no spraying had higher pest infestation than *C. juncea* plots sprayed with Attack. Attack was thus, likely responsible for the low pest numbers. *Crotalaria juncea* was not a good candidate for habitat manipulation for conservation biological control compared with the remaining five plant species.

Pests encountered in the present study are among the most destructive herbivores that cause problems to brassica production worldwide. *Plutella xylostella*, the most damaging among them is difficult to manage globally^39,40^. *Hellula undalis* and *B. brassicae* also pose serious challenge to brassicas crops^41^. For decades, these pests have been managed with the use of synthetic insecticides resulting in various ecological concerns^42,43^. Suppression of cabbage pests with natural enemies has been found to give better pest control, high and quality crop yield compared with application of synthetic insecticides^7,37,44^. A study on cabbage root fly *Delia radicum* (Diptera: Anthomyiidae) showed a reduced number of pupae and larvae of the pest in habitat manipulation treatments compared with the no manipulation control^45^. Another experiment that assessed impact of flower strips on cabbage moth *Mamestra brassicae* L. (Lepidoptera: Noctuidae) and cabbage white butterfly, *Pieris rapae* L. (Lepidoptera: Pieridae) Pfiffner *et al*.^46^ found that parasitism of *M. brassicae* and predation of *P. rapae* were enhanced in flower strip treatments compared with the no flower strip control.

Botanicals too have been successful against pests of brassica crops e.g.^22–24,32^. Concurrent use of conservation biological control with botanical insecticides may result in better pest suppression compared with each of them in isolation^30^. Habitat manipulation and botanical insecticides played a complementary role in achieving pest suppression. The potential of each plant in supporting natural enemies for conservation biological control and as botanical insecticide is therefore key to maximising the potential benefits of dual pest management services. Habitat manipulation with *C. juncea* plus Attack resulted in an effective suppression of pests compared with habitat manipulation with *C. juncea* and sprayed with extract of *C. juncea*. On the other hand, habitat manipulation with *A. conyzoides* sprayed with either Attack or extract of *A. conyzoides* both resulted in effective pest suppression and in some cases the aqueous extract spray option performed better than the Attack.

Pests suppression in this study highlights the potential role of predators in conservation biological control. Predators have been acknowledged to effectively suppress pests in managed agroecosystems^7,47^ even though parasitoids have often been the focus of biological control of brassica crop pests^31,48^. Spiders and ladybird beetles were generally more numerous in the habitat manipulation plus botanical insecticides and habitat manipulation plus tap water treatments compared with habitat manipulation plus Attack treatments indicating the potential negative impact of Attack on predators. A study in Nicaragua found that plots sprayed with synthetic insecticides had low numbers of generalist predators including spiders compared with a no insecticides application treatment^7^. The current study therefore supports the notion that aqueous plant extracts are relatively harmless to natural enemies^49–51^ and can be integrated with biological pest control unlike synthetic insecticides such as Attack which has deleterious effects on natural enemies. This insecticide (Attack) is a semi-synthetic derivative of the natural product abamectin in the avermectin family^52,53^. It is considered to have minimal effect on predators and parasitoid because it degrades rapidly through sunlight on the leaf surface resulting in shorter contact periods in the crop environment^52^. Some studies have found Attack^®^ less harmful to natural enemies than other synthetic insecticides and therefore recommend its inclusion in integrated pest management programmes e.g.^51,54,55^. Other studies have, however, found it to have negative impacts on natural enemies e.g.^32,56–58^. In assessing the acute and sub-lethal toxicity of 14 pesticides on the generalist predator *Orius laevigatus* (Hemiptera: Anthocoridae), Biondi *et al*.^59^ found Attack to be moderately harmful until seven days after spraying. Khan *et al*.^57^ and Biondi *et al*.^59^ cautioned that the inclusion of insecticides such as Attack in IPM programmes should carefully be evaluated. In the present study, treatments sprayed with Attack had very low numbers of spiders and ladybird beetles.

Numbers of predators were higher in the no habitat manipulation sprayed with tap water (control) compared with the habitat manipulation treatments where floral resources and shelters were available. This might have happened because natural enemies moved between plots and showed aggregative numerical responses to where prey were most abundant. Monsrud and Toft^60^ reported that, aggregative numerical response occurs when predators respond to increasing number of prey or are attracted to alternate prey that aggregate around honeydew produced as a result of aphid feeding. In a three-year study on effect of floral resources on cabbage root fly *D. radicum*, Nilsson *et al*.^61^ found that while there was an overall increase in hymenopteran parasitoid catches in habitat manipulation treatments, parasitism was higher in the control treatment where pest infestation was higher compared with the habitat manipulation treatments. The study further explained that parasitoids may feed on floral resources but also needed prey to complete their life cycle, hence, the high parasitism rate in areas with high pest infestation. Donaldson *et al*.^62^ reported that numbers of predators increased in response to increasing population of soybean aphid and decreased when aphid population decreased. In the present study, aggregative numerical responses could have occurred as there was high *B. brassicae* infestation in the no plant habitat manipulation treatment.

The various plant species showed differences in supporting conservation biological control resulting in differences in crop yield. Borders of *T. triangulare* gave higher cabbage yields compared with, for example, *C. juncea*. Floral morphology, colour and duration of bloom may affect the potential of various plant species to support the activities of natural enemies for pest suppression as reported by Anderson and Dobson^63^ and Begum *et al*.^64^. *Talinum triangulare* for instance, is a perennial species with bright floral colours and persistent blooming and might have attracted natural enemies more effectively than other plant species. This result indicates that plant species selection is vital to maximising outcomes in conservation biological control interventions^19^.

Generally, there was improvement in yield and quality of cabbage season after season. This might be due to enhanced local densities of predators and possibly local suppression of pests. Similar results have been reported when cabbage pests were managed using IPM practices in which high marketable yields were obtained as a results of enhanced natural enemy activities^7,44^. In season one, yields of each habitat manipulation treatment sprayed with either botanical insecticides or Attack resulted in higher yields compared with habitat manipulation without spraying. However, in subsequent seasons, yield in some of the sole conservation biological control treatments were higher than treatments with both conservation biological control and spraying. This could be due to an additive effect of conservation biological control and spraying on cabbage yield in season one and trade-offs in latter seasons. Trade-offs between biological control and the application of insecticides have been reported^7,44^. Habitat manipulation for conservation biological control could be complemented with the application of biological pesticides such as botanical insecticides during the early stages of the season when numbers of natural enemies may not be enough to keep pests below what could cause economic injury to crops. Activity of natural enemies cannot, however, be ruled out in season one as all habitat manipulation treatments outperformed the no habitat manipulation treatments in both yield and quality.

Though efforts were made to reduce inter-plot interference, the 5 m alley between treatments was small compared with the movement patterns of agricultural arthropods that can readily move meters. Consequently, treatments that are close to each other might have had effect on each other. However, the marked differences in yield and quality among the various habitat manipulation with plant treatments and the no plant manipulation as well as treatment with various spraying options indicate that the closeness of the plots did not entirely mask the potential of each of the habitat manipulation or spraying treatments.

Yields observed in the present study compare favourably with those obtained in similar studies using the same crop variety at the same site e.g.^32,33^ and in the same locality e.g.^51^ indicating that habitat manipulation for conservation biological control alone or in combination with botanical insecticides have the potential to effectively manage brassica crop pests.

In this study, habitat manipulation sprayed with tap water had the lowest cost of protection while habitat manipulation sprayed with Attack had the highest cost. For a pest management tactic to be adopted, output in terms of crop yield and quality and income from the sale of produce must be appreciable enough to offset the cost of protection and result in profit. It was observed that irrespective of the high cost of protection for habitat manipulation plus spraying treatments (both botanical and Attack) they had higher net incomes compared with their corresponding habitat manipulation without spraying treatments in season one. This was the result of a high yield and quality in all habitat manipulation plus spraying treatments compared with treatments with only habitat manipulation. But in seasons two and three most of the habitat manipulation plus tap water and habitat manipulation plus botanicals recorded higher incomes than the habitat manipulation and Attack treatments. This may have resulted from the overall improvement in yield and quality of the habitat manipulation plus tap water and habitat manipulation plus botanical treatments.

Incomes were generally higher in season one for all treatments than in season two because prices of undamaged cabbage heads were 25% higher in season one than in season two. However, incomes in season three were higher than the two previous seasons even though the price of cabbage heads was the same as in season two. This was due to an increase in cabbage yields in season three compared with season two. In addition, there were no damaged yields in season three, meaning all yields attracted the normal market value thereby demonstrating that profitability is a factor of cost of production, quality and quantity of yield as well as the market value of produce at any given time^65,66^.

Cost: benefit ratios were higher in season one and declined towards season three. This was because income from the control treatment was lowest in season one and increased towards season three. This was due to enhanced yield and quality of cabbage which might have resulted from possible movement of natural enemies from habitat manipulation plots to control plots (Griffith^45,67^). Cost: benefit ratios in this study were calculated based on income from the control treatment hence, increase in yield and income of the control treatment reduced the cost: benefit ratio.

Cost: benefit ratios show the economic viability and biological effectiveness of pest management interventions in relation to the control^66,68^. Positive ratios denote economic viability whilst negative ratios mean the pest management tactic was not cost-effective. The highest cost: benefit ratio obtained in this study is superior to those attained in other studies while the lowest is below those obtained in those studies e.g.^65,66,69^. In all these studies, only cost of plant protection was used in calculating the ratios, and income from the control was the reference point.

In the present study, cost: benefit ratios ranged between 1: −2.23 for habitat manipulation with *T. procumbens* sprayed with Attack (TriAttack) in season three up to 1: 49 for Talwater in season one. While some treatments were strongly beneficial in all seasons, others were only beneficial in some seasons. NopAttack had cost: benefit ratios of 1: 33.50, 1: 9.81 and 1: 4.64 for seasons one, two and three respectively whereas Triwater had cost: benefit ratios of 1: 28.50, 1: 23.60 and 1: 17.84 for seasons one, two and three respectively. On the hand, TriAttack had cost: benefit ratios of 1: 20.04 and 1: 14.03 in season one and two respectively and 1: −2.23 in season three being negative, suggesting that combination of two pest management tactics may have additive effects resulting in positive cost: benefit ratios in early seasons but trade-offs may occur in latter seasons. In such instances, combination of tactics should only be encouraged when it is absolutely necessary to achieve optimum and cost-effective pest suppression.

## Conclusions

The combination of habitat manipulation for conservation biological control and application of botanical insecticides can result in effective pest suppression with corresponding increase in crop yield, quality and income. The use of synthetic insecticides has always been a curative attempt to salvage crops from damage. However, it often leads to elimination of important organisms in the agroecosystem. Selection of insecticides to use alongside habitat manipulation should be done carefully to avoid negative impacts on natural enemies that are fundamental to successful conservation biological control. Similarly, the choice of plant species for habitat manipulation should cautiously be considered because not all plant species have the potential to provide floral resources for natural enemies. Further care should be taken if dual ecosystem services of botanical insecticides and conservation biological control are to be achieved. There is the possibility of trade-off between the provision of floral resources and insecticidal activity by the same plant species. There is evidence that compounds that have activity against insects may be found in the nectar of some plant species. In the current study, habitat manipulation plus tap water was more cost-effective than habitat manipulation plus spraying with botanical insecticides or Attack^®^. Combination of other compatible tactics with conservation biological control should be done only when it is indispensable to achieve ideal pest suppression. In such cases, inexpensive and ecologically benign insecticides such as botanicals should be preferred over synthetic insecticides such as Attack^®^.

## Materials and methods

### Location of experiments

Experiments ran over three consecutive seasons of cabbage *Brassica oleracea* var. *capitata* (Brassicaceae) production at the Crops Research Institute (CRI), Kwadaso, Kumasi, Ghana (6°43′N,1°36′W; 287 m asl) between January 2017 and March 2018. The three seasons were between June and August 2017 (major rainy season), September and November 2017 (minor rainy season) and December 2017 and March 2018 (dry season). The site was in the wet, semi-deciduous forest zone of Ghana with average rainfall of 1,484 mm in 137 wet days per annum. Average temperature is 25.6 °C in the range of 19.6 °C and 35 °C and average relative humidity of 83.2%.

### Experimental design and treatments

Experiments consisted of a 7 × 3 factorial in a randomized complete block design with split plot arrangement and replicated four times. The main plot factor consisted of seven levels of habitat manipulation i.e. with six non-crop plant species and a no plant (control) i.e. 1. *A. conyzoides* 2. *T. procumbens* 3. *C. juncea* 4. *C. citratus* 5. *L. camara* 6. *T. triangulare* and 7. no plant (bare earth control) (Table 8). The sub-plot factor was three levels of spraying: 1. Botanical insecticide prepared from the same plant species as bordering the crop of that plot, 2. Conventional insecticide (Attack^®^, emamectin benzoate) as positive control, and 3. Negative control (tap water) (Table 8). Main plots measured 11 m × 3 m with 5m-wide bare earth alley between plots. Each main plot was divided into three sub-plots (3 m × 3 m) to accommodate the three levels of spraying with a 1m-wide bare earth alley between subplots. The ‘no plant’ habitat manipulation treatment did not have a botanical sub-plot treatment, thus there was a total of 20 treatments and 80 plots.

**Table 8:** Treatment combination of plant species for habitat manipulation and spraying and abbreviated treatment identifier.

Treatment (crop border*spraying)	Botanical	Attack^®^	Water
*Ageratum conyzoides*	Agerabot	AgeraAttack	Agerawater
*Crotalaria juncea*	Crotbot	CrotAttack	Crotwater
*Cymbopogon citratus*	Cymbot	CymAttack	Cymwater
*Lantana camara*	Lanbot	LanAttack	Lanwater
*Talinum triangulare*	Talbot	TalAttack	Talwater
*Tridax procumbens*	Tribot	TriAttack	Triwater
No plant (no border)	—	NopAttack	Nopwater

Note: bot = Botanical, Attack = emamectin benzoate.

Water = tap water, − = no sub-plot treatment.

Habitat manipulation plants were established in the field between January and May 2017 to reach blooming stage (except *C. citratus* which does not flower) before transplanting of cabbage. Cabbage *B. oleracea* var. *capitata* (cv. Oxylus) seedlings were grown for five weeks after which they were transplanted to the field at a spacing of 0.5 m × 0.5 m. For the conservation biological control treatment with no border plant, seven rows of seven cabbage stands were planted per sub plot. Other treatments had six rows of seven cabbages per sub plot with the relevant border plant as a central strip with three rows of cabbage either side. This resulted in 42 plants per sub plot (49 for the control). Experiments were kept free from weeds by hand hoeing. Well decomposed poultry manure at 500 g per cabbage plant was applied 10 days after transplanting to enhance soil fertility. The experiment was rain-fed but manual irrigation was done when required, especially during the dry season.

### Spraying and data collection

Each main plot had three sub-plots that received the three spraying levels i.e. botanical, Attack and tap water. Aqueous extracts of the plants were used at concentration of 10% w/v. Matured leaves of each of the six plants were collected from around the experimental area and weighed when fresh and blended using kitchen blender. Leaves were not collected from the habitat manipulation plots so as not to disturb natural enemies. In all cases, blending was done until no plant fragments were visible to the naked eye^22^. Mixtures were sieved using muslin cloth to obtain a particle-free extract and was transferred into a 15 L capacity Knapsack sprayer for immediate application^32^. A 1 ml Sunlight liquid soap per litre of plant extract was added to the aqueous extracts prior to spraying. Attack was used at the recommended rate of 1.5 ml per litre. Spraying was done weekly beginning from three weeks after transplanting cabbage seedlings and continued for four consecutive weeks in each season.

Numbers of pests including diamondback moth *Plutella xylostella* L. (Lepidoptera: Plutellidae), cabbage aphid *Brevicoryne brassicae* L. (Hemiptera: Aphididae) and cabbage webworm *Hellula undalis* Fab. (Lepidoptera: Crambidae), and natural enemies including spiders (Araneae) and ladybird beetles (Coccinellidae) were assessed weekly, three weeks after transplanting cabbage seedlings and a day before spray application for four consecutive weeks. Four plants were randomly selected in each sub-plot to count pest and natural enemy numbers. Numbers of *B. brassicae* were often difficult to count so were scored on a scale of 0–5 (0 = no infestation and 5 = highest infestation i.e. large continuous colonies)^32^.

### Economic analysis

#### Field establishment of non-crop plants for habitat manipulation

The number of minutes used in gathering and planting the conservation biological control border plants for each plot was recorded and extrapolated to assume a commercial crop area of one hectare. Values varied according to how easily each was acquired and established. At the time of the study, one person-day at the location of the experiments costed US$ 8.80. *Lantana camara* was established using stem cuttings and required nursing before field establishment so 11 person-days were calculated to be required to establish *L. camara* for a cabbage crop area of one hectare resulting in US$ 96.80. *Cymbopogon citratus* established easily, but it was relatively difficult to get the propagules due to its popular use as anti-malaria herb in Ghana. Therefore, 10 person-days were required to gather and establish costing US$ 88.00. Seedlings were used to establish *C. juncea* and to ensure good stand, replacement of dead seedlings was required, hence 10 person-days at US$ 88.00 were required to establish a crop area of one hectare. *Ageratum conyzoides* and *T. procumbens* were the most abundant weed species around the experimental area and were relatively easy to gather so 8 person-days valued at US$ 70.40 for each. *Talinum triangulare* was the easiest to establish but was relatively difficult to gather due to its use as an indigenous vegetable in Ghana. Therefore, 9 person-days at US$ 79.20 was required to establish it. For these border plant treatments, the cost of plant protection was solely the cost of collecting and establishment.

#### Estimating cost of spraying

In each of the seasons, there were four spray applications of aqueous extracts and emamectin benzoate (Attack) to the respective sub plots and the costs of these interventions were extrapolated to assume a commercial crop of one hectare. Simple knapsack sprayers are used on farms in Ghana so 3 person-days were required to spray one hectare of cabbage. Thus, 12 person-days (amounting $ 105.60) were required for the four spray applications per season. The no habitat manipulation sprayed with Attack treatment required, 3.2 person-days to spray one hectare. Thus, 12.8 person-days at $112.64 was required for each season. The difference was due the higher plant stand compared with the habitat manipulation treatments. Collecting plant materials and preparing aqueous plant extracts for each habitat manipulation and botanical treatment required 2 person-days per hectare. Cost of volume of Attack required to spray one hectare was $15.00. Thus, for each season $60.00 of Attack was required to spray the no habitat manipulation plus Attack treatment whilst $55.00 worth of Attack was required to spray the habitat manipulation plus Attack treatments. The reduction in the amount required for Attack was due to the reduced plant stand in the habitat manipulation plus Attack treatments. Sunlight liquid soap at $1.00 for each habitat manipulation and botanical insecticide treatment per season was used. Thus, cost of plant protection for habitat manipulation and spraying treatment combinations = cost of establishing non-crop plants + labour cost of collecting and preparing botanical extracts + labour cost of spraying + cost of sunlight liquid soap. For habitat manipulation sprayed with Attack, cost of protection = cost of establishing non-crop plants + cost of Attack + labour cost of spraying. For no habitat manipulation sprayed with Attack, cost of protection = Cost of Attack + labour cost of spraying.

### Computing cost: benefit ratios

Total income was obtained by adding incomes from both undamaged and damaged heads. Income from undamaged yield was obtained by multiplying the head yield per hectare by the selling price per kg of cabbage head. Income from damaged heads was obtained by multiplying damaged head yield by selling price per kg of damaged heads. Net benefit per hectare for each treatment was derived by subtracting the total cost of plant protection from total income of each treatment. Benefit over the no habitat manipulation and spraying control was obtained by subtracting the income of the control treatment from that of each habitat manipulation or habitat manipulation + spraying treatment. The cost: benefit ratio of each treatment was derived by subtracting the income of the control treatment from the net income of each treatment and the products were divided by total cost of plant protection for each treatment^65^.

### Yield assessment and marketing of cabbage heads

Cabbage heads in each treatment were separated into undamaged and damaged heads and weighed. Undamaged heads were without visible signs of caterpillar feeding or holes whereas damaged heads had visible signs of insect feeding, but had reduced market value. The local currency, Ghana cedi (ɇ) exchange rate to the US$ was 1:0.22 during the period of the study and this exchange rate was used to calculate values in US$. At the first season harvest, 1 kg undamaged cabbage head was sold for US$ 0.44 and damaged was US$ 0.22 at the local (Agric Nzema) market. At the harvest of both second and third seasons, price per kg of undamaged and damaged heads were US$ 0.33 and US$ 0.22, respectively. Income from the sale of cabbage was converted to per hectare by extrapolating plant population of the habitat manipulation treatments to 35,000 plants per hectare assuming planting distances of 0.5 m × 0.5 m and considering the area occupied by the non-crop plants for manipulation and spaces for easy movement while the no habitat manipulation treatments had 35,500 plants per hectare considering only spaces for easy movement.

### Statistical analysis

Mixed model repeated measure analysis was performed using the statistical package for social scientists (SPSS) IBM version 24 (IBM, 2016). Where significant differences were observed (i.e. *P*-value <0.05), mean separation was done using Tukey’s *post-hoc*, Honestly Significant Difference (HSD) test.
